# Sob as marcas de bexigas: varíola e africanos na Corte imperial na primeira metade do século XIX

**DOI:** 10.1590/S0104-59702026000100034

**Published:** 2026-10-26

**Authors:** Tânia Pimenta, Flavio Gomes

**Affiliations:** i Pesquisadora, Casa de Oswaldo Cruz, Fundação Oswaldo Cruz, Fiocruz. Rio de Janeiro – RJ – Brasil. tania.pimenta@fiocruz.br; ii Professor, Universidade Federal do Rio de Janeiro. Rio de Janeiro – RJ – Brasil. escravonovo@gmail.com

**Keywords:** Escravidão, História da varíola, Rio de Janeiro, Africanos, História da saúde, Slavery, History of smallpox, Rio de Janeiro, Africans, History of health

## Abstract

O artigo identifica o impacto da varíola sobre africanos e seus descendentes no Rio de Janeiro na primeira metade do século XIX a partir de diferentes modos de aproximação: registros de óbitos; anúncios de fugas, venda e roubo; e discursos médicos sobre a doença. Argumentamos que, embora a varíola não tenha aparecido como a enfermidade com maior evidência entre as causas de morte de escravizados, esteve presente para os médicos e para aqueles que sobreviviam à doença. As sequelas, em grande parte em forma de marcas na pele, constituíam um sinal da escravidão alardeado nos anúncios de fuga, roubo ou venda.

## Abstract (en)

This article identifies the impact of smallpox on Africans and their descendants in Rio de Janeiro in the first half of the nineteenth century, drawing on different approaches: death registry; announcements of escapes, theft or sale; and medical discourses on the disease. Our argument is that although smallpox did not appear as the most evident infirmity among the enslaved people’s cause of death, it was present for doctors and those who survived it. The sequelae, mostly in the form of marks on the skin, were a sign of slavery highlighted in announcements of escapes, theft or sale.

No anúncio de 22 de novembro publicado no *Diário do Rio de Janeiro* de 1839 aparecem notícias do africano Felizardo, de “nação Moçambique”. Tinha escapado dois meses antes, em 19 de setembro. Dele se sabia: “explica-se muito quando fala, de sorte que parece crioulo” e que “consta andar passeando sempre pela bica dos Marinheiros, comendo e bebendo por aquelas vendas”. Aos “Senhores Pedestres”, havia a recomendação expressa para que tivessem a “bondade de fazer toda a diligência, e ver se o agarram, pois que serão bem recompensados” aqueles que “o apanhar[em] e levar[em] a Rua do Senado entre a do Espírito Santo, e Lavradio, casa sem número, pegada ao número 7”. E seguia um alerta: “Se protesta com todo o rigor da lei contra aquela pessoa que o acoitar em sua casa” (Diário do Rio de Janeiro, 1821-1841, [seção Escravos fugidos, 22 nov. 1839, p.4]).

Para além das tópicas sobre comportamentos, linguagens e mesmo prováveis paradeiros, o anúncio oferecia descrições físicas, ajudando possíveis denúncias, localização e captura. Assim, Felizardo tinha “estatura ordinária, rosto comprido”, trazia “uma bicha na orelha” e o mais importante: era “bastante picado de bexigas”. Fundamentais “sinais” para a sua rápida localização, não obstante a informação de que “muda de traje todos os dias”.

Fossem marcas de bexigas, como era denominada popularmente a varíola, fossem de vacinação, a identificação desses sinais pelas pessoas em geral era possível e necessária porque, durante a primeira metade do século XIX, a varíola fazia parte do cotidiano dos habitantes do Brasil e, em especial, da população de escravizados na cidade do Rio de Janeiro. A varíola esteve intimamente ligada à história do comércio atlântico de homens e mulheres africanos escravizados. Ela acompanhou milhares de pessoas deslocadas compulsoriamente de diversos sertões do continente africano, fosse pelas migrações forçadas para fugir da doença e das secas, fosse pela captura e comércio que facilitaram a sua disseminação devido às aglomerações nos barracões no litoral, às viagens nos porões dos chamados navios negreiros e ao desembarque em diversos portos com a imediata disponibilização para compra e venda (Alden, Miller, 1987).

Neste artigo, abordamos algumas implicações, marcas e repercussões da varíola nas experiências de africanos atingidos por ela, incluindo a mortalidade e os vacinados, no Rio de Janeiro na primeira metade do século XIX. Para um exercício empírico, avaliamos diversos modos de nos aproximarmos da varíola e do seu impacto sobre as populações urbanas de escravizados africanos, a partir dos registros de sepultamentos e anúncios de fugitivos publicados nos jornais. Outros caminhos surgiram a partir de simulações com a demografia africana e os seus efeitos na mortalidade, embora os números da morte tenham escondido mais do que revelado aspectos da varíola na cidade do Rio de Janeiro. Também recorremos aos médicos – no caso aos seus discursos – que atuavam na Corte e como eles associavam o desenvolvimento de epidemias, não só de varíola, com as travessias atlânticas e a incorporação reiterada e massiva de escravizados africanos.

O enfrentamento contra a varíola contava com um recurso único na época: a vacina. Apresentada em 1798, na Inglaterra, pelo médico Edward Jenner, a vacina antivariólica de origem animal era baseada no pus produzido pelos bovinos acometidos pelo *cowpox*. Essa vacina – deixando apenas uma marca onde havia sido inoculada – protegeria de manifestações severas da varíola que se desenvolvia em todo o corpo, reproduzindo “sinais” e marcas permanentes.

A historiografia considera que a vacina tenha sido introduzida no Brasil em 1804, embora o estudo de Camargo (2007) centrado em São Paulo indique 1803.^1^ Pouco depois da chegada da corte portuguesa, em 1808, ao Rio de Janeiro, foi criada a Junta da Instituição Vacínica pelo alvará de 4 de abril de 1811. Após a independência política, em 1822, observa-se que a instituição da vacina e sua disseminação estavam inseridas no processo de estruturação do Império. Em 1846, com o decreto de 17 de agosto, o governo imperial ampliou e centralizou o serviço no Instituto Vacínico do Império, com a missão de “estudo, prática, melhoramento, e propagação da vacina”. Tratava-se de um serviço de saúde com objetivo de ampliar o seu alcance com a interiorização da vacinação. Desse modo, o Estado enviava vacinadores para os municípios colocando-se presente no território, ainda que houvesse grande precariedade, tanto devido à falta de remuneração, ou a seu baixo nível, concedida aos vacinadores como às dificuldades de obtenção e do transporte do pus vacínico (Pimenta, Barbosa, Kodama, 2019, p.338).^2^

É preciso, porém, considerar que a vacina antivariólica deve ter coexistido por décadas com a variolização. Essa prática era bastante antiga e utilizada em vários pontos do mundo, baseada na introdução de cascas trituradas ou pus de feridas de alguma pessoa variolosa.^3^ O problema é que não era raro o desenvolvimento da doença em sua forma agressiva a partir da variolização (Chalhoub, 1996, p.106; Fernandes, 2010). Alden e Miller (1987, p.210) apontam que o mundo colonial português começou a recorrer mais à variolização a partir da década de 1790. De fato, circularam diversas cópias de uma carta de 9 de julho de 1799, endereçadas a todos os governadores ultramarinos, com ordem do príncipe regente para que procurassem introduzir a inoculação de “Meninos Negros e Índios, visto ter mostrado a experiência ser este o único e eficaz preservativo contra o terrível flagelo das Bexigas naturais, que tem causado tão consideráveis estragos nas Colônias Portuguesas” (Visconde de Anadia, 1 out. 1802a). Ordenava-se ainda que, “por meio dos Médicos, e das Casas dos Expostos onde as houver, e com exemplo e persuasão, procure fazer adotar a prática de inoculação, e dê conta dos progressos que se fizerem neste importante objeto”, conforme a cópia enviada pelo visconde de Anadia (1 out. 1802a) para Francisco da Cunha e Menezes, governador da Bahia. Essa cópia é datada de 1º de outubro de 1802, assim como a que foi enviada para Fernando Antônio de Noronha, governador de Angola (Visconde de Anadia, 1 out. 1802b).

Um ano e meio depois, Noronha recebia outra carta do mesmo visconde, discorrendo sobre o tema, com o objetivo de promover o convencimento dos moradores de Angola a seguir o exemplo de Moçambique, onde, de acordo com o relato do próprio governador,

há tanto conhecimento da inoculação e da sua utilidade que esta prática é muito usual e que estão os seus habitantes tão familiarizados com ela que uns e outros se inoculam depois do que principiam a sentir as bexigas, mesmo trabalhando sem experimentarem mal efeito pois que de cada 100 inoculados, apenas morre um, e que ultimamente se observou que o capitão de um navio francês inoculou com a vacina duzentos e cinquenta e seis negros, de que constava a carregação, e que só lhe morreu um, e que finalmente todos os carregadores ali inoculam a sua escravatura (Visconde de Anadia, 26 abr. 1804).

Conforme investigações de Roquinaldo Ferreira, os acervos africanos – em especial o Arquivo Histórico Nacional de Angola – guardam substantivos registros sobre as ações do Império português para combater a disseminação das bexigas. Também governantes coloniais diversos – além das autoridades portuguesas – estimulavam a variolização, num primeiro momento, e a vacina, a partir de 1805 pelo menos. É o que aparece na carta do visconde de Anadia (8 jun. 1805) para o governador de Angola, Fernando de Noronha, avisando que a Coroa havia decidido enviar o “pus vacínico” com o novo cirurgião geral destacado para Luanda. Fica evidente – entre outros aspectos – a confiança depositada pela Coroa e pelos traficantes na vacinação contra a varíola visando limitar as perdas de escravizados, fossem no litoral africano, na travessia e no imediato desembarque. Em 1819, o governador de Angola, Manoel Vieira de Albuquerque Tovar, enviou um ofício ao Senado da Câmara de Luanda determinando que todos os escravizados fossem imunizados com a vacina. A esse respeito, um capitão informou que todos os cativos que embarcaram em seu navio haviam sido vacinados, assim como os habitantes de Luanda (Tovar, 6 nov. 1819).

No ano seguinte, o mesmo governador escreveu para o mandatário de Benguela, Mathias Joaquim de Brito. Garantia que a Coroa, em aviso de 20 de março de 1820, havia aprovado o uso de vacinas nos escravizados, recomendando que o número de vacinados aumentasse. Determinava ainda um responsável pela vacinação e orientava que, caso as vacinas não surtissem efeito, enviasse alguns rapazes para Luanda para que fossem vacinados lá e transportassem para Benguela o pus vacínico (Tovar, 3 ago. 1820). Tovar faz referência a um ofício de Brito, confirmando o recebimento da vacina (“um vidro”) para ser aplicada nos escravizados, além dos 16 que estariam em observação. Ainda assim, julgando a importância de tais questões, Tovar (5 dez. 1820) solicitava informações detalhadas, estranhando tanta demora e tanto silêncio.

Em abril de 1826, o então governador de Angola, Nicolau de Abreu Castelo Branco (8 abr. 1826), enviou uma carta para o físico-mor do Reino, ordenando que ele e o cirurgião-mor se revezassem na inspeção dos escravizados “em atenção a estarmos na estação mais perigosa e de mais trabalho”.^4^ Havia a preocupação em minimizar o efeito de epidemias que pudessem se disseminar na região e em outras partes do império português. A documentação sobre a vacinação em áreas africanas no primeiro quartel oitocentista sugere que tais recomendações podem ter contribuído para a diminuição da mortalidade por varíola tanto nos portos africanos quanto naqueles em várias partes do Brasil, como apontam Alden e Miller (1987, p.213). Em 1831, com a legislação de proibição ao tráfico, os médicos que analisavam os impactos das epidemias e endemias passaram a debater ainda mais as conexões entre a entrada massiva de africanos e o desenvolvimento de epidemias. A variável espacial – cidades portuárias no Brasil – e a ilegalidade do tráfico tornavam ainda mais difíceis a realização de inspeções sanitárias.

Surtos de determinadas doenças e o seu caráter epidêmico eram sempre associados à intensificação – apesar da ilegalidade – do comércio atlântico de africanos. Justificativas médicas, morais e econômicas – assim como desfaçatez, obscurantismo e interesses – esgrimavam argumentos, apontando tanto para a intensidade da entrada ilegal e massiva de africanos como para o conluio da sociedade escravista e a explosão epidêmica. Entre as enfermidades com maior incidência sobre os africanos se destacavam, além da varíola, a tísica pulmonar, o tétano, a pleurisia crônica, a hipoemia intertropical e o maculo. Outras moléstias se apresentavam com surtos frequentes e eram associadas ao tráfico atlântico: disenteria, oftalmia e sarampo (Sigaud, 2009 [primeira edição em 1844]; Rego, 1872).

## Epidemias de pensamentos: os médicos, a varíola e o tráfico

No século XIX, o Rio de Janeiro e seus arrabaldes permaneceram como os principais locais de desembarque de africanos escravizados. Ao mesmo tempo – segundo Pereira Rego^5^ –, a população carioca enfrentou aproximadamente cinco ciclos epidêmicos de varíola ainda na primeira metade do Oitocentos, sendo os mais extensos e letais os que ocorreram entre 1834 e 1836, prolongando-se até a primeira metade de 1837. Essas recorrentes epidemias suscitaram reflexões sobre as formas de disseminação da varíola, assim como sobre o acesso à vacina e as condições do comércio de escravizados. Boa parte dos médicos da época depositava muita esperança na difusão da vacinação para eliminar a varíola (18ª Sessão, nov. 1835, p.9-17).

Um debate público ocorrido na sessão de 6 de dezembro de 1834 da Sociedade de Medicina do Rio de Janeiro (SMRJ)^6^ ajudou a fomentar as críticas acerca do comércio de africanos escravizados, principalmente pela ilegalidade. Alguns médicos e cirurgiões defendiam abertamente a ideia de que as bexigas tinham sido e continuavam sendo importadas pelos africanos. Doutor Meirelles^7^ explicitava que o “tráfico continuava a ser feito, e fazia-se ainda mais do que antigamente em todo o litoral”, acrescentando que “a epidemia tinha se desenvolvido em muitos negros novos, e principalmente nos da Casa de Correção, onde havia deles um grande depósito”. Além disso, Meirelles anotou como muitos vacinados, e “alguns com cicatrizes vacínicas” bem caracterizadas, tinham sido infectados por bexigas (18ª Sessão, nov. 1835, p.11-12). Sigaud^8^ destacou como, em 1834 e 1835, a varíola reapareceu no Rio de Janeiro depois de cinco anos sem se manifestar, ainda que a doença tivesse se desenvolvido “primeiramente entre os jovens moleques recentemente desembarcados e recebidos na casa de correção” (Sigaud, 2009, p.150). Acrescentou que na “cidade como nos hospitais, os indivíduos já vacinados, portadores de cicatrizes das pústulas da vacina, foram igualmente afetados, mas em número menor do que aqueles que não tinham nunca tomado a vacina” (p.150). Interessante é como Pereira Rego analisou os registros da epidemia de 1834. Ele concordava que o tráfico ilegal de africanos – cada vez mais acentuado – determinava a explosão epidêmica da varíola: “Muitos africanos aqui chegados com o flagelo, como tivemos ocasião de ver, eram clandestinamente introduzidos para o seio da nossa população, ou alojados em depósitos nos subúrbios da cidade, é mais razoável acreditar que fosse por eles importada” (Rego, 1872, p.23).

Se a epidemia de 1834 era “atribuída à importação de escravos eivados dessa afecção, que aqui foram clandestinamente introduzidos” (Rego, 1872, p.63), no ano seguinte a varíola persistia caracterizada como “de mau caráter e causando muitos falecimentos” (p.10), ao lado de outras doenças. Para os períodos posteriores, a enfermidade continuaria marcando presença, embora o caráter de gravidade apenas retornasse no início de 1837, atacando vacinados e não vacinados, “levando o estrago e a destruição a todos os pontos e às freguesias de fora, e só veio a extinguir-se em dezembro” (p.23). Novos surtos de varíola no Rio de Janeiro surgiram entre 1844 e 1848. Para 1847, Pereira Rego (1872, p.50) destacou que, mesmo considerando a curta duração e pouca intensidade, a explosão da contaminação era “resultado da importação de numerosos africanos dela eivados, que chegaram nesse ano, e foram espalhados pelo centro e arredores da cidade. E de feito nenhuma moléstia há que mais vezes nos fosse trazida pelo tráfego [*sic*] de escravos, e que mais devastações causasse nesta cidade e nas fazendas do interior”.

Os debates e publicações médicas sugerem pensar em consensos em torno da vacina, embora houvesse uma ou outra discussão sobre a sua capacidade de proteção completa ou mesmo sobre a expectativa de que a doença pudesse se desenvolver de forma branda. Para Sigaud (2009, p.151), o surto epistêmico de 1834 e 1835, “apesar de ter poupado em grande parte os que tinham sido vacinados, matou, todavia, alguns deles, mas a maioria deve à vacina o fato de ter sido preservado ou atingido de forma benigna”. Ele se referiu a João Alves Carneiro, um “prático experimentado”, corroborando o argumento sobre o papel importante da vacina no enfrentamento da varíola. Carneiro foi além da observação dos efeitos da vacinação no Rio de Janeiro, defendendo a visão de que a cessação da varíola no início da década de 1830 não era resultado da “não introdução de negros pela via do contrabando, mas isso sim da precaução tomada no litoral da África pelos traficantes, que consistia em vacinar os negros antes de embarcá-los, a fim de evitar o desenvolvimento da varíola durante a travessia e as terríveis consequências da mortalidade: esta precaução tinha dado os mais felizes resultados” (p.150-151).

A partir de investigações pioneiras, inclusive sobre os enterramentos de cativos no cemitério da Misericórdia, Karasch (2000) considerou que a campanha contra a varíola enfatizava a vacinação de africanos ainda em Angola ou das crianças e os “pretos novos” no imediato desembarque no Rio. Assim, os próprios traficantes acabaram por perceber que os vacinados sobreviviam mais à travessia. Embora os anúncios de venda de escravizados indicassem aqueles já vacinados, visando maior valorização da negociação deles, não faltaram denúncias de que capitães de navio, negociantes, mercadores e senhores eram negligentes quanto à vacinação, a despeito da disponibilização de vacinas na Corte. A própria Karasch (2000, p.215-216) observou – ao analisar dados de 1848, quando a entrada de africanos pode ter alcançado quase sessenta mil, enquanto as vacinações registradas somaram cerca de 2.300 – baixa taxa de imunização pela vacina. De qualquer modo, estudos para o Caribe apontam a piora das condições de saúde ao longo da travessia com a ilegalidade do tráfico Atlântico (Barcia, 2020). As destacadas investigações de Dale Graden (2014) mostraram os impactos das epidemias e doenças articulando-se com o comércio atlântico em Cuba e Brasil.

Para a primeira metade do Oitocentos importa destacar que as comunidades médicas estavam se organizando em torno de instituições como a Faculdade de Medicina do Rio de Janeiro^9^ e a SMRJ, além de periódicos especializados em medicina. Foi também um período de institucionalização da medicina acompanhada da elaboração de uma agenda sanitária baseada no higienismo, na qual se destacava a correlação entre climas e doenças no pensamento médico (Ferreira, 1999, 2009; Edler, 2011). Escritos médicos registraram polêmicas acerca das origens de doenças, das terapias consideradas adequadas, dos diagnósticos convincentes e das formas de disseminação num contexto no qual os preceitos neo-hipocráticos explicavam tanto a emergência de enfermidades quanto as práticas terapêuticas. Alimentos, roupas, comportamentos e ventilação, juntamente com as características climáticas, eram importantes elementos que influenciavam o surgimento de enfermidades, a ocorrência de epidemias, suas causas, formas de controle e ações sanitárias.

Todas essas questões eram consideradas relevantes no pensamento médico de então em torno da varíola. Debates na SMRJ também avaliavam os tratamentos disponíveis para os variolosos. Na sessão de 5 de maio de 1832, foram apresentados observações e relatos da experiência do cirurgião formado, João Alves de Moura, sobre o uso de um óleo “mui eficaz para favorecer, e promover a erupção das pústulas variolosas, quer nos casos de erupção incompleta, ou viciada, quer nos de repercussão” (Sobre o uso..., 1838, p.5). O importante era a defesa de que os remédios inventados e praticados por leigos poderiam ter alguma eficácia e não deveriam ser desprezados de antemão. Sobre a terapêutica, Moura garantia que, “ainda que por ora não possa dar explicação do remédio, nem de seu modo de obrar” (p.7), os seus efeitos se mostraram “pronunciados, prontos, constantes e maravilhosos” (p.7). Para Moura, a não erupção das bexigas era causa de muitas mortes; assim, o remédio, de aplicação externa, era bastante seguro. Embora o cirurgião não tivesse a receita de preparo do tal óleo, deduzia que o ingrediente principal eram lagartixas brancas, emendando comparações com a vacina de Jenner e a utilização da quina, baseadas em empirias próprias. Justificava sua dedução com suas experiências no uso de determinados compostos e diferentes óleos. Naquele momento pretendia apenas apresentar de modo simples as observações feitas durante a aplicação do remédio, cuja receita fora recolhida por um velho boticário que vendia o óleo sem revelar a sua composição (Sobre o uso..., 1838; cf. Lima, 2011). Interessa aí destacar a origem do “remédio” e os alvos nas aplicações de alguns daqueles testes, recebendo o tratamento com o óleo: um preto com cerca de 14 anos de idade de um negociante; uma preta e um preto de uma “partida de escravos” do negociante F. Aguiar; um preto de 16 anos; um escravo de um proprietário de uma fábrica de cera; um preto do dono de um trapiche.

Os casos em que o óleo foi aplicado se concentravam quase todos em escravizados e africanos, o que suscita mais reflexões sobre a temática da varíola e escravidão africana urbana no Rio de Janeiro. A clientela de Moura era constituída basicamente de escravizados? Ou os bexiguentos tratados por ele eram, em sua maioria, escravizados? Ou o cirurgião considerava mais adequado experimentar o óleo em escravizados africanos? De todo modo, trata-se de um registro de assistência médica voltada para escravizados na primeira metade do Oitocentos no Rio de Janeiro. Interessante é que, anos depois (em 1844), Sigaud (2009, p.151) se referiu ao uso do óleo tirado do corpo de lagartixas brancas “experimentado com sucesso” por Moura, acrescentando que os práticos brasileiros tinham “vigilância ativa para prevenir” algumas consequências da varíola. Sigaud observara que, nas epidemias, a doença podia progredir para a confluência das pústulas, o que dava aparência de pele de *chagrin* (pele de lixa), que seria um sintoma mortal na maioria dos casos. Outro problema que poderia decorrer das sequelas da varíola era a cegueira. Sigaud anotou como era incalculável o número de “negros que ficaram cegos em consequência da varíola” (p.151).

## Percentuais de sofrimento e cálculos da dor

Numa atmosfera de numerosos desembarques de africanos, batizados coletivos, expectativas comerciais, ajuntamentos compulsórios no Lazareto, inspeções sanitárias e demandas por sepultamentos em massa, surgem registros sobre as enfermidades que grassavam nos navios do comércio atlântico, despejando terror e medo. Exalavam odores da morte e perfumes das sobrevivências, além de desejos de vida. A demografia atlântica exibiria muitos mortos, mas escondeu epidemias, silenciou enfermidades e os seus perfis fúnebres. Realizamos incursões metodológicas em registros de sepultamentos em diferentes fontes cliométricas, tentando encontrar se não a varíola, ao menos as suas vítimas.

Um primeiro olhar se origina de uma amostragem de sepultamentos de escravizados africanos registrados entre 1801 e 1830 para a paróquia da Candelária, área central carioca (Livro…, 1801-1830). Na amostra que consideramos há registros de 2.050 sepultados, sendo 1.074 adultos e 976 inocentes. Entre os adultos há 782 africanos, representados por 496 homens e 286 mulheres, indicando os padrões de gênero do comércio atlântico com 63,5% africanos do sexo masculino. Do total de 437 africanos com causa das mortes determinadas, localizamos 104 (24%) escravizados – qual seja, quase 1/4 de sepultados – indicando “por bexigas” na *causa mortis*.

**Tabela 1:** : Escravizados sepultados por bexigas, Candelária, 1801-1830

Africanos	Crioulos	Total
**Adultos**		
36 (92,3%)	3 (7,7%)	39 (37,5%)
**Inocentes**		
1 (1,5%)	64 (98,5%)	65 (62,5%)

Fonte: Arquivo da Cúria Metropolitana do Rio de Janeiro, Livro de óbitos, paróquia da Candelária.

Há equilíbrio entre africanos e nascidos no Brasil, com ligeira predominância de crioulos e mais equilíbrio entre homens (49%) e mulheres (51%). Entre os 37 africanos com identificações de nomenclaturas de “nações” determinadas se destacam benguelas, com 19,5%, e cabindas e moçambiques, com 15,4% cada. Considerando as faixas etárias dos africanos sepultados por varíola – nos registros de óbitos da paróquia da Candelária –, temos 9% na faixa de até 10 anos de idade, portanto crianças, 54,5% deles entre 11 e 20 anos e 36,5% acima de 20 anos.

Uma grande expectativa nossa era encontrar os impactos de mortalidade e epidemias em sepultamentos de africanos que aparecem nos registros de “pretos novos” para a paróquia de Santa Rita entre 1812 e 1829 (Livro…, 1812-1814, 1824-1829).^10^ Os sepultamentos nos períodos de 1812-1818 e 1825-1829 perfazem cerca de 11.500 escravizados – parte considerável de africanos recém-chegados – na Corte imperial, em pleno funcionamento do porto do Valongo.^11^ Para o período inicial de 1812-1818 há escassas informações sobre as causas da mortalidade. Localizamos 43 registros com identificação mais detalhada, sendo 88,5% desses “por bexigas”. Para o período de 1825-1829 a falta de informações sobre a causa da mortalidade é ainda maior. Não há fartos detalhes sobre os padrões de mortalidade – embora não se tenha dúvida de que esses sepultamentos incorporavam a base africana recém-desembarcada, ou seja, indivíduos mortos a bordo ou no período imediatamente posterior ao desembarque. Assim, é possível avançar sobre a relação de mortos com os principais portos de origem dos embarques. Para o período de 1812-1818, temos a informação de que 73% daqueles que morreram por bexigas, cujos portos de embarque eram conhecidos, teriam vindo de Angola, Cabinda, Luanda e Moçambique. Para os registros de 1825-1829 há informações mais generosas sobre africanos sepultados com indicações daqueles que morreram a bordo, qual seja, durante a travessia. De 3.758 registros de sepultamento de africanos, 743 destacavam que as mortes tinham acontecido a bordo ou no imediato desembarque. Há indicações que concentram as procedências (49,3%) em regiões de Angola e portos de Luanda, seguidas pela área sul da África Central (25,6%).

Outra amostra para localizar impactos do tráfico atlântico e a mortalidade por varíola são os registros de encaminhamento de enterramentos da Santa Casa de Misericórdia em 1835, 1836, 1839, 1842 e 1845. Em análises em andamento, estamos verificando padrões de mortalidade, doenças e enfermidade para cerca de 9.200 escravizados, sendo 90% deles africanos. Considerando os registros com *causa mortis* identificadas, temos uma amostra de 4.773 registros, dos quais 194 referiam-se a mortes por bexigas (aparecem na maior parte como “bexigas confluentes” e “bexigas malignas”), perfazendo 4% apenas. Provenientes de Angola, Congo, Cabinda e Benguela perfazem 46,7% dos infectados por varíola segundo a identificação “bexigas” nesses registros.

Ainda podemos recorrer aos registros de internação no Hospital da Santa Casa da Misericórdia apresentados por Pereira Rego para 1837. A varíola podia apresentar caráter grave, que frequentemente levava à morte, ou caráter benigno, quando os sintomas eram mais leves. O próprio médico exemplifica a diferença de evolução entre as duas formas ao relatar que observou no Hospital da Misericórdia, em 1837, 143 doentes, dos quais 41 eram casos varioloides e de varicela. Dos 102 restantes de varíola, 46 eram de benigna e 56 de confluente, dos quais 48 morreram (Rego, 1872, p.22-23). Assim, o número de mortos em decorrência da varíola entre africanos e seus descendentes era bastante alto quando se desenvolviam as bexigas confluentes. Contudo, de acordo com os médicos, a mortalidade era modificada pelo número de vacinados. Quando a vacinação contava com um bom pus vacínico e com bons vacinadores, em geral, a varíola não se desenvolvia ou mostrava-se em sua forma benigna.

Para além da vacinação na Corte – que poderia talvez explicar números não tão expressivos de mortes por varíola –, ao analisarmos os dados quantitativos de causas de morte de escravizados, devemos considerar a possibilidade de que essa informação não tenha merecido muita atenção de quem produzia tais documentos. O estudo de Daniel Oliveira (2018) sobre os registros de óbitos de Porto Alegre da segunda metade do século XIX aponta para o cuidado maior nas informações relacionadas a pessoas brancas, o que pode ser ponderado para o contexto da Corte da primeira metade do Oitocentos em relação aos escravizados. As Posturas da Câmara Municipal, de 1830, estabeleciam na “primeira seção” sobre saúde pública que um sepultamento deveria ser feito com a apresentação de uma “Certidão do Facultativo, que o houver assistido, na qual este declare a enfermidade, de que morreu, e a sua duração, assim como a hora da morte, e a morada do falecido; no caso, porém, de não ter havido assistente, ou de a morte ter sido repentina, o respectivo Juiz de Paz nomeará um Facultativo para ir examinar o dito Corpo” (Continuação..., 11 dez. 1830).

Como já verificamos, quase metade dos registros tinha descrição sumária sem a causa da morte, como o da “preta Rosa Benguela, escrava da Sra. D. Antonia Thereza de Jezus”, no qual constava apenas “pode sepultar-se” em 17 de fevereiro de 1835, de acordo com um inspetor da freguesia de Santana (Registros..., 17 fev. 1835). Além disso, a morte por varíola implicava enterros rápidos, o que pode ter contribuído para o sub-registro.

Outra questão que merece ser mencionada refere-se à concomitância e à sequência de epidemias de outras doenças como escarlatina ou febre reumática eruptiva, por exemplo. Epidemias de varíola também eram acompanhadas de surtos de sarampo e coqueluche. Durante as décadas de 1830 e 1840, esse processo de organização dos registros das doenças ainda estava sendo padronizado (Pimenta, 2024), e, caso o enfermo apresentasse outros problemas de saúde, sobretudo considerando-se as muitas epidemias que grassavam no período, talvez parte das mortes por varíola tenha sido registrada como causada por outra enfermidade. Soma-se a isso o possível pouco cuidado com os registros de morte dos escravizados, embora a varíola tivesse sintomas bastante característicos. Além disso, importa destacar que os dados do cemitério da Santa Casa registram apenas uma parte das mortes de cativos, uma vez que muitos acabaram sendo enterrados em florestas e quintais de seus donos ou despejados no mar e abandonados em terrenos baldios (Karasch, 2000, p.144).

Estes pontos – registro de mortos sugerindo outras enfermidades, pouco cuidado com as indicações da causa da morte de escravizados e enterros não declarados oficialmente – podem ajudar a refletir sobre o descompasso entre a importância dada pelos médicos à varíola e à vacinação no cotidiano e o pouco destaque dessa doença observado na mortalidade registrada nos enterramentos do cemitério da Santa Casa e outros assentos de óbitos que mostramos. Essas considerações devem ser somadas ao avanço da vacinação na Corte do Rio de Janeiro, conforme é colocado por Karasch (2000, p.215), avaliando o pequeno número de mortes que indicaria “um controle melhor da doença na cidade, como resultado de programas de vacinação”. Chalhoub (1996, p.110, 112) afirma que a vacina deve mesmo ter tido importância no controle da varíola, mas aprofunda a análise na Corte ao longo do tempo, mostrando que, “pelo menos no que concerne aos escravos”, os anos 1820 tiveram serviço de vacinação mais bem sucedido do que os anos 1840.

Ainda que houvesse problemas quanto à continuidade e à qualidade do serviço de vacinação na Corte, as dificuldades eram muito maiores pelo interior do Brasil. Apesar disso, constituía-se uma estrutura voltada para a saúde pública ao longo do século XIX. A epidemia de febre amarela de 1849 e 1850, contudo, é considerada um momento de inflexão no modo como o Estado passou a lidar com a saúde pública, criando um órgão centralizado, a Junta de Higiene Pública,^12^ para melhorar o estado sanitário da cidade do Rio de Janeiro e de outras povoações do Império. Diante dos registros de outras epidemias, de fato, a febre amarela se destacou por ter causado 4.160 mortes na Corte, num contexto em que se debatia sobre o fim do tráfico atlântico de africanos e se constatava que a doença atingia mais imigrantes e viajantes estrangeiros (Chalhoub, 1996, cap.2). Poucos anos depois, em 1855 e 1856, a epidemia de cólera também provocou muitas mortes no Rio de Janeiro: 5.228, considerando-se as freguesias de fora em ambos os casos (Rego, 1873, p.40, 110). Os africanos foram, proporcionalmente, mais atingidos pela epidemia (Kodama et al., 2012), o que deve ter contribuído para o medo entre a população e a preocupação entre os senhores de escravos durante a epidemia.

No início da década de 1870, refletindo sobre a epidemia em questão, o então presidente da Junta entre 1864 e 1881, José Pereira Rego (1873, p.109), concluía que, embora o cólera tenha inspirado terror devido aos relatos vindos da Europa e dos EUA, não teria sido muito mortífero, e que a doença havia feito mais estragos entre “os pretos, os homens de cor e as classes mais inferiores da sociedade, mas foi entre os mendigos que ela se mostrou mais violenta”. Explicitava-se, assim, que a vulnerabilidade e as mortes dos africanos e seus descendentes eram consideradas de importância menor do que as de pessoas brancas.

## Anúncios endêmicos entre marcas e sinais

A varíola sobressaía nos discursos médicos, multiplicava-se na correspondência oficial, mas escapava dos registros de sepultamentos e causas de mortes. Ainda sabemos pouco sobre o impacto cotidiano da varíola na população de escravizados – e menos ainda entre os africanos – na Corte do Rio de Janeiro na primeira metade do século XIX. Não há dúvida de que a varíola estava ali em corpos e mentes de africanos, entre memórias, dor, marcas, sinais, crenças, curas e medos. Um caminho foi verificar os anúncios de escravos fugidos e aqueles de compra e venda de africanos escravizados, onde proliferaram narrativas corpóreas. A seção “Escravos fugidos” ocupava considerável espaço entre os anúncios publicados nas principais folhas da Corte durante o século XIX. A partir da descrição física e de determinados comportamentos do cativo, além de outras informações, buscava-se ajuda para localizá-lo e, muitas vezes, oferecia-se recompensa pela captura. Qualquer detalhe podia auxiliar na identificação de quem havia fugido; portanto, doenças aparentes ou suas sequelas também seriam importantes para o reconhecimento. Assim, não eram raras as referências às marcas de bexigas.

Para além de especificar se o rosto ou qualquer parte do corpo era muito ou pouco picado de bexigas, era possível indicar se as marcas eram recentes ou antigas. Desse modo, em 1836, foi anunciado no *Diário do Rio de Janeiro*: “Fugiu de uma Chácara no Cosme Velho, um preto de nome Manoel, nação Congo, idade 20 anos, levou vestido calça e camisa de algodão americano, estatura ordinária, com sinais de bexigas ainda de fresco, de 3 a 4 meses, e olhos meio espantados; quem dele souber e der notícia na rua da Misericórdia, n.216, receberá alvíssaras” (Diário do Rio de Janeiro, 1821-1841, [seção Escravos fugidos, 14 mar. 1836]). Quem anunciou estava certo de que essa informação era importante e de que os pedestres, em geral, seriam capazes de identificar sinais recentes de bexigas. Ou de vacinação, de acordo com os seguintes anúncios de 1834 e de 1837, respectivamente: “Desapareceu da casa da rua do Lavradio, sobrado da esquina da de Rezende n.1, um moleque de nome João, nação Angola, terá 12 a 14 anos de idade, reforçado do corpo, foi vacinado há pouco, e conserva ainda nos braços as cicatrizes da vacina, e levou calças de riscado; dão-se boas alvíssaras a quem o apreender” (Diário do Rio de Janeiro, 1821-1841, [seção Escravos fugidos, 13 jan. 1834]). Ou na seção de “Roubos”, quando se publicou: “Furtaram no dia 24 do corrente, da rua do Piolho n.51, um moleque de nome Joaquim, nação Monjolo, idade 15 anos, alto, mui direito, e magro, rosto meio redondo, e riscado nas faces, beiços, e nariz finos, é bem escuro, e estava vacinado haverá 3 dias: dá-se 100$ rs. de gratificação a quem o trouxer à casa acima” (Diário do Rio de Janeiro, 1821-1841, [seção Roubos, 26 set. 1837]).

Marcas de bexigas ou de vacina ajudavam, portanto, a identificar um fugitivo entre os milhares de cativos que circulavam pela cidade. Além disso, constituíam um aspecto valorizado por quem comprava um escravizado, uma vez que significava que não seriam acometidos pela enfermidade em novos surtos ou, ao menos, que desenvolveriam uma forma benigna da doença. No caso das crianças, isso também era bastante importante, dada a alta taxa de mortalidade. Muitas vezes desprezados como registros da invenção de corpos e almas africanas, os anúncios dos jornais revelam muito mais do que a insubordinação. Olhares senhoriais traduziam corpos africanos, intenções físicas e gestos mentais, identificando valores e sentidos da escravidão urbana e os impactos do comércio de escravizados, atravessados por controle, proibição, ideologias e debates públicos e privados.

Não poucos anúncios expunham contextos de vulnerabilidade: “Vende-se no sobrado n.42, rua da Quitanda, 1 aparelho de porcelana para jantar, com 177 peças, 1 cadeirinha muito leve, 1 relógio de cima de mesa, e 1 lindo crioulinho de 9 meses, que come de tudo; já engatinha, foi vacinado, já teve sarampo, e catapora, e está muito forte e gordo” (Diário do Rio de Janeiro, 1821-1841, [seção Vendas, 6 fev. 1836]). Na mesma lista do aparelho de jantar e do relógio de mesa, o bebê era anunciado à venda sem indicações sobre o motivo. Não se sabe se sua mãe havia morrido ou se a intenção era mesmo separá-lo dela. O que fica evidente é que as informações sobre a saúde pareciam ser relevantes para esse anúncio de venda de 1836. Já havia sido vacinado contra a varíola, tinha tido sarampo e catapora, portanto, seria uma compra com menos riscos de perda porque dificilmente desenvolveria alguma forma grave dessas doenças. Cinco anos depois, outro anúncio vendia a mãe com sua filha,^13^ destacando que a bebê já era vacinada com 4 meses:

Amas de Leite – Vende-se uma excelente ama de leite, parda, de 20 anos, com uma linda cria fêmea de 4 meses, muito clara, bonita, e muito gordinha, já batizada e vacinada de bexigas; o leite é muito bom, e a rapariga é de muito boa conduta, e muito carinhosa para crianças, sabe engomar perfeitamente, cozinhar, lavar, cozer, e todo o mais serviço interno de uma casa; na rua de S. Pedro n.180 (Diário do Rio de Janeiro, 1821-1841, [seção Escravos fugidos, 15 nov. 1841]).

Desse modo, tais registros também indicam que a vacinação era considerada eficaz por, ao menos, parte dos proprietários de escravizados. Assim, seguimos nomes e trajetórias daqueles que conviveram com a varíola, sendo marcados por ela em seus corpos, via infecção, vacinação e cura. Para a Corte imperial, em centenas de anúncios de fugas, aluguel, e compra e venda de escravizados localizamos corpos, marcas, medos, precauções, cálculos, picadas, estigmas e experiências de inoculação sobre a varíola em descrições, tópicas e imagens. Doenças, marcas de mortes e cura aparecem e desaparecem, sempre fugidias como as mentes e os comportamentos dos cativos que se evadiam. Descrições e sinais sobre a infecção e a vacinação da varíola revelam práticas, expectativas, percepções sobre doenças, epidemias, transmissão e as conexões com as margens africanas, de comércio e experiências atlânticas.

Apresentamos uma abordagem qualitativa com uma amostra de anúncios de fuga (somente consideramos os africanos) publicados no *Diário do Rio de Janeiro* entre 1821 e 1841. Nessa amostragem de quinhentos anúncios verificamos inúmeras descrições associadas a “bexigas” e destacamos alguns no Quadro 1 com o objetivo de exemplificar os diversos tipos de anúncios.

**Quadro 1:** : Registros sobre “bexigas” nas descrições dos africanos em anúncios de fugidos no *Diário do Rio de Janeiro*, 1821-1841

“grande costura de bexigas entre os olhos”	“bastantes sinais de bexigas”	“escapo das bexigas”	“alguma coisa picado de bexigas”
“sinais de bexigas na cara”	“na testa uma cicatriz das mesmas bexigas”	“picado de bexigas no peito”	“marcas brancas das mesmas bexigas”
“sinais de bexigas no nariz”	“tendo já bexigas”	“poucos sinais de bexigas”	“é marcado de bexigas”
“cara cheia de bexigas”	“bonito de cara, porém cheia de borbulhas que parecem bexigas”	“por estar tratando de moléstia de bexigas”	“sinais de bexigas que mal se percebem”
“com três sinais de bexigas vacinadas em ambos os braços”	“com nariz crivado de bexigas”	“ainda não teve bexigas”	“tendo ainda os sinais frescos da vacina das bexigas”
“muito bexigosos por ter acabado há pouco tempo de ter bexigas”	“com dois sinais de enxerto de bexigas no braço e os abraços”	“fula com marcas de bexigas”	“cara com bexigas”
“livre de bexigas”	“sinais de bexigas grandes no rosto”	“sinais de bexiga nos beiços”	“sinal como de bexigas no queixo direito”

Fonte: Diário do Rio de Janeiro, 1821-1841.

“Bastante picado de bexigas”, “sinais de bexigas”, “marcas de bexigas” referiam-se, muito provavelmente, às sequelas deixadas pela doença, pois uma pessoa que enfrentava a varíola dificilmente teria forças para se movimentar pela cidade, além de ter menos chance de conseguir quem a amparasse. De qualquer modo, abordagens qualitativas apontam para tópicas corporais, nas quais doenças, enfermidades, comportamento, origem africana e experiência da escravização na Corte eram associados. Em anúncio de julho de 1822 foi registrada a escapada do africano José, “um preto Moçambique”, listando na sua descrição fisionômica a seguinte ordem dos “sinais seguintes”: era “muito picado de bexigas”, tinha “nas fontes e mesmo na testa sinais da mesma Nação” e era “muito grosso, e muito reforçado” (Diário do Rio de Janeiro, 1821-1841, [seção Escravos fugidos, 1 jul. 1822]). Já Catharina, “de Nação Caçasse”, apresentava “estatura ordinária, bem preta, cara comprida, e com sinais de bexigas, cabelo cortado”. Detalhe: “O maior sinal que tem é ter leite pois criava uma filhinha, que teve o desamor de deixar e fugir” (Diário do Rio de Janeiro, 1821-1841, [seção Escravos fugidos, 5 dez. 1823]). No ano seguinte – mais precisamente em agosto –, era vez de anunciar o desaparecimento de “Manoel, de Nação Benguela, idade de 18 anos, alto, e proporcionalmente de corpo, bem preto, rosto comprido, algum tanto beiçudo nariz rombudo bons dentes sinais de bexigas na cara”. Interessante é que era “bastante ladino” e tinha “vindo a pouco de Minas” (Diário do Rio de Janeiro, 1821-1841, [seção Escravos fugidos, 7 out. 1824]). Também com “sinais de Bexigas” se escafedeu “um preto, oficial de Pedreiro, Congo de Nação, chamado André” com a “fala branda e vagarosa” (Diário do Rio de Janeiro, 1821-1841, [seção Escravos fugidos, 1 jul. 1825]).

Numa quarta-feira do alvorecer de junho de 1828 era publicada a fuga coletiva de quatro africanos pertencentes a Manoel Peixoto da Costa Lonzada, morador na rua do Sabão, número 328. As descrições de “bexigas” só aparecem para dois deles, no caso Manoel, de nação Congo, oficial de pedreiro, que era “picado de bexigas, com validas em ambos os olhos”, tendo “falto dos dentes de diante, alguma cousa baixo, com os peitos um tanto saídos para fora”, e também João, de nação Libollo, que tinha “estatura ordinária, com poucos sinais de bexigas”. Para os outros dois africanos não havia referências sobre enfermidades, sendo “João, Nação Cabinda, meio fulo” e “Antônio, Nação Songo, bem feito de corpo, e de pernas, cor retinta, sem ponta de barba, os olhos alguma cousa rasgados” (Diário do Rio de Janeiro, 1821-1841, [seção Escravos fugidos, 4 jun. 1828]). Para a africana Maria foi necessário tanto descrevê-la como sendo Moçambique, entre 18 e 20 anos, mas também “de boa estatura, bastante reforçada e gorda, cara redonda, nariz chato, picada das bexigas, com riscos da Nação nas faces”, assim como por ter “nas costas e braços sinais antigos de castigo”. Dela se sabia “estar acoitada em casa dos parentes; e já tem sido vista no Campo da Aclamação, por Valongo, Mata Porcos, e Glória” (Diário do Rio de Janeiro, 1821-1841, [seção Escravos fugidos, 9 out. 1829]). Quem escapou de uma chácara em São Cristóvão foi “um preto novo, de Nação Inhambane, com marca de picos miúdos desde a testa até a ponta do nariz, é rapaz, bem parecido, grosso do corpo, pernas alguma cousa finas e arqueadas, estatura ordinária, acabado a pouco de ter bexigas naturais, que ainda manqueja quando anda por ter as solas dos pés alguma cousa dolorida” (Diário do Rio de Janeiro, 1821-1841, [seção Escravos fugidos, 3 abr. 1829]). Comprado há pouco tempo, quem fugiu da casa de José Gonçalves de Amorim, um tenente ajudante do Segundo Regimento de Infantaria da Segunda Linha da Corte, foi o africano Anastácio, de nação Quilimane. Acabaria descrito como “ainda boçal”, tendo desaparecido “indo ao Campo buscar água”. Nas descrições desse africano aparentemente recém-comprado se detalhou que tinha “idade 14 anos pouco mais ou menos, magro, não é bonito, nem elegante no olhar, cor escura, pés secos; reparando bem vê-se que tem um dos dedos mínimos dos pés menor do que o outro, tem no cachaço um sinal pouco vulgar, e nas costas outro bem visível de quando teve bexigas (diz ele)”, sugerindo as traduções corporais feitas pelos próprios africanos para explicar marcas e sinais do seu corpo sob os olhares senhoriais (Diário do Rio de Janeiro, 1821-1841, [seção Escravos fugidos, 17 jun. 1829]). Com “sinais de bexigas na cara”, além de “baixo, gordo e bem barbado”, o fugitivo “Francisco, nação Angola, idade 55 a 58 anos” tinha currículo de insubordinação. No alvorecer de 1840 foi registrada a sua escapada – em dezembro de 1839 –, pois “em poder do outro senhor já fez uma longa fugida de quatro anos, sabendo sempre escapar a vigilância das autoridades, calçando-se, já mudando o nome e a voz, e finalmente fazendo todas as artimanhas próprias de mestre naquele ofício”. Tem mais: “Consta estar refugiado nos subúrbios da cidade de Niterói, onde foi apreendido na última fugida” (Diário do Rio de Janeiro, 1821-1841, [seção Escravos fugidos, 18 jan. 1840]). Francisco só perdia para um fugido “escravo de Dona Camilla Amália Rebello de Azevedo, moradora nesta cidade”. Na véspera do Natal de 1840 seu nome nem sequer foi registrado no anúncio de fuga. Talvez não fosse necessário, pois “acha-se fugido há seis anos”. Sendo de “nação Nagô”, tinha “estatura ordinária, cheio de corpo, olhos muito vivos, tem nas faces riscas (sinais de sua nação), bastantes cicatrizes de bexigas no nariz, as maçãs do rosto levantadas, e queixo fino”. Tinha também os sinais de muita esperteza, pois “faz uso de uma carta de alforria falsa, que furtou de um preto Moçambique; consta que vive no porto da Estrela negociando em criação” (Diário do Rio de Janeiro, 1821-1841, [seção Escravos fugidos, 4 maio 1841]).

## Considerações finais

Talvez nunca saibamos se Felizardo conheceu maiores alegrias na sua escapada – ao menos se divertindo, “comendo e bebendo”. Contudo, foi possível acompanhar que seu senhor publicou o anúncio de fuga mais algumas vezes, com pequenas variações acerca das características físicas, roupas e locais onde havia sido visto ao longo de 1839. As marcas que as bexigas deixaram em sua pele, certamente, o acompanharam pela vida e serviram para identificá-lo. Não sabemos também se ele sobreviveu à varíola por ter se submetido à variolização ou por ter sido vacinado em Moçambique antes de embarcar ou logo ao chegar no Rio de Janeiro, como aconteceu com muitos. Tampouco podemos afirmar que Felizardo tenha recebido tratamento médico durante o padecimento, o que significava uma oportunidade para muitos médicos, inclusive os mais renomados, para experimentar medicamentos ou adquirir mais experiência.

A partir da documentação analisada – anúncios de fuga, compra e venda; debates médicos sobre as epidemias de varíola, o tráfico atlântico e a vacina; e registros de óbitos – podemos apontar que a varíola constituía, no período investigado, mais uma marca da escravidão nos corpos de africanos e seus descendentes. Embora, em termos quantitativos, a varíola tenha aparecido com menor frequência entre as causas de morte, a historiografia e a investigação empírica que apresentamos mostram que suas cicatrizes eram visíveis. Para os médicos, representavam sinais de adoecimento e sobrevivência; para os senhores, indícios de propriedade e instrumentos de identificação; para os próprios cativos, marcas que se somavam às dores do cativeiro e às demais inscrições corporais, como as marcas de nação.

Esses sinais, portanto, não eram apenas indicação de uma doença superada, mas testemunhos das condições a que africanos e seus descendentes eram submetidos. Nas aglomerações dos navios, nos depósitos urbanos e nos espaços de trabalho, a disseminação da varíola encontrava terreno fértil, transformando a pele dos sobreviventes em testemunho das violências do tráfico e do regime escravista. Ao lado de outras marcas – cicatrizes, feridas, tatuagens ou inscrições forçadas –, as sequelas da varíola eram mobilizadas em anúncios de fuga, imagens, registros e descrições médicas, atestando a experiência da escravidão.

## Data Availability

Não estão em repositório.

## NOTAS

1 Sobre variolização, introdução da vacina jenneriana e tratamento dos variolosos entre escravizados do sul dos EUA, ver Savitt (2002). A respeito do Caribe, ver Sheridan (2009) e Brizola (2022).

2 A respeito da institucionalização do serviço de vacinação, além de Chalhoub (1996) e Fernandes (2010), ver Portugal (2018).

3 Sobre a variolização entre sociedades africanas e a relação dessa prática com crenças religiosas, ver Chalhoub (1996) e Soumonni (2012).

4 Em 1808, a Real Junta do Protomedicato foi substituída pela Fisicatura-mor, sendo instituídos os cargos de físico-mor e cirurgião-mor. Nesse ano, a corte e as instituições do império português foram transferidas para o Rio de Janeiro, onde permaneceram até a independência política do Brasil, em 1822. Assim, após esse período, as instituições portuguesas voltaram a ser sediadas em Portugal, e suas colônias voltaram a se dirigir a Lisboa para tratar de questões relacionadas à saúde pública e ao exercício das artes de curar.

5 O médico José Pereira Rego foi presidente da Junta Central de Higiene Pública entre 1864 e 1881 e da Academia Imperial de Medicina entre 1855 e 1857 e 1864 e 1883. Em 1874, recebeu o título de barão do Lavradio.

6 Fundada em 1829, foi substituída pela Academia Imperial de Medicina em 1835.

7 Joaquim Cândido Soares de Meirelles foi um dos fundadores da Sociedade de Medicina do Rio de Janeiro, cirurgião-mor da Armada e membro da Junta Central de Higiene Pública.

8 Joseph François Xavier Sigaud (1796-1856), médico francês que atuou no Rio de Janeiro de 1825 a 1856, usava o nome aportuguesado em suas publicações. Publicou *Do clima e das doenças do Brasil* (1844), foi um dos fundadores da Sociedade de Medicina do Rio de Janeiro, em 1829, e editor de alguns periódicos especializados, como *O Propagador das Ciências Médicas* e o *Diário de Saúde.*

9 Em 1808, foi criada a Escola de Cirurgia no Rio de Janeiro (decreto de 2 de abril de 1808), transformada em Academia Médico-Cirúrgica (decreto de 1º de abril de 1813) e, posteriormente, em Faculdade de Medicina do Rio de Janeiro (lei de 3 de outubro de 1832).

10 Com financiamento do projeto Proep/CNPq/COC-Fiocruz, realizamos a produção de bancos de dados dos registros de óbitos da Paróquia de Santa Rita para os períodos de 1812-1814 e 1824-1829. Assim contabilizamos todos os registros diretamente das fontes. As investigações pioneiras com esses dados apareceram em Pereira (2007, 2024).

11 Para o Valongo, ver as investigações mais sistematizadas nos estudos de Soares (2023, 2018 e 2017). Nem sempre recebendo os créditos devidos e as citações correspondentes, a base da sua pesquisa encontra-se no relatório do seu pós-doutorado em arqueologia (Soares, 2013).

12 A Junta foi criada pelo decreto de 14 de setembro de 1850. O seu regulamento foi publicado com o decreto de 29 de setembro de 1851, renomeando-a Junta Central de Higiene Pública.

13 Sobre maternidade e infância sob a escravidão no mundo atlântico, ver Carula, Ariza (2022) e os dossiês organizados em *Slavery & Abolition* e *Women’s History Review* por Cowling et al. (2017, 20, 2018).
